# Supplementary material for: Weight Change is Associated With Metabolic Liver Health in a General Population Extending Beyond Weight Loss Targets of International Guidelines
Source: Gastro Hep Adv. 2025 Oct 10;5(2):100831. doi: 10.1016/j.gastha.2025.100831 (PMC12670896; doi:10.1016/j.gastha.2025.100831)
Supplement: Supplementary Material [file mmc1.pdf]

Weight change is associated with metabolic liver health in a general population extending beyond weight loss targets of international guidelines

Laurens A. van Kleef[1], Mesut Savas[2,3] *, Maurice Michel[4,5] *, Cyrielle Caussy[6,7,] Jesse Pustjens[1],

Adriaan G. Holleboom[8,9], Elisabeth F.C. van Rossum[2,3], Harry L.A. Janssen[1,10], Jörn M. Schattenberg[4,5] and Willem P. Brouwer[1]

* Indicates shared authorship

1. Department of Gastroenterology and Hepatology, Erasmus MC, University Medical Center, Rotterdam, The Netherlands
2. Department of Internal Medicine, Division of Endocrinology, Erasmus MC, University Medical Center, Rotterdam, The Netherlands
3. Obesity Center CGG, Erasmus MC, University Medical Center, Rotterdam, The Netherlands
4. Department of Internal Medicine II, Saarland University Medical Center, Homburg, Germany
5. Saarland University, Saarbrücken, Germany
6. Hospices Civils de Lyon, Département Endocrinologie, Diabète et Nutrition, Hôpital Lyon Sud, 69495 Pierre-Bénite, France
7. Univ Lyon, CarMen Laboratory, INSERM, INRA, INSA Lyon, Université Claude Bernard Lyon 1, 69495 Pierre-Bénite, France
8. Department of Vascular Medicine, Amsterdam UMC, The Netherlands.
9. Amsterdam Gastroenterology Endocrinology and Metabolism Institute, Amsterdam UMC, The Netherlands
10. Toronto Centre for Liver Disease, Toronto General Hospital, University Health Network, Canada

SUPPLEMENTARY DOCUMENTS

Supplementary Table 1: Participant characteristics

	≥3 % weight loss	stable weight	≥3% weight gain
n	1877	2957	1968
**Demographics**			
Age	50 [33, 63]	53 [38, 64]	40 [26, 56]
Male	919 (49.0)	1576 (53.3)	830 (42.2)
Ethnicity			
Asian	174 (9.3)	479 (16.2)	213 (10.8)
Black	536 (28.6)	717 (24.2)	552 (28.0)
Hispanic	450 (24.0)	599 (20.3)	522 (26.5)
Other	129 (6.9)	117 (4.0)	105 (5.3)
White	588 (31.3)	1045 (35.3)	576 (29.3)
**Comorbidity**			
Weight status (1-year prior)			
BMI ≥ 30 kg/m^2	1081 (58.5)	929 (31.9)	553 (28.6)
BMI 25-30 kg/m^2	505 (27.3)	981 (33.7)	602 (31.2)
BMI < 25 kg/m^2	262 (14.2)	998 (34.3)	776 (40.2)
Diabetes	440 (24.3)	540 (19.0)	240 (12.6)
Hypertension	936 (53.1)	1480 (52.9)	782 (42.4)
High waist circumference	1112 (60.9)	1523 (52.6)	1231 (63.9)
**Biochemistry**			
AST	18 [15, 22.25]	19 [16, 24]	19 [16, 24]
ALT	17 [13, 24]	18 [13, 26]	18 [13, 27]
HDL	1.4 (0.4)	1.4 (0.4)	1.4 (0.4)
Triglycerides	1.2 [0.9, 1.8]	1.3 [0.9, 1.9]	1.2 [0.9, 1.9]
**Outcomes**			
MASLD	774 (41.2)	1246 (42.1)	850 (43.2)
At-risk MASH	112 (6.4)	162 (5.9)	140 (7.6)
LSM ≥ 8 kPa	166 (9.2)	262 (9.1)	179 (9.3)
CAP	263 (62)	264 (62)	266 (62)
LSM	5.1 [4.2, 6.2]	5.0 [4.1, 6.1]	4.9 [4.0, 6.1]

Data is presented as mean (SD), median [P25-P75] or n and percentage.
Abbreviations: ALT, alanine aminotransferase; AST, aspartate aminotransferase; CAP, controlled attenuation parameter; HDL, hypodensity lipoprotein; LSM, liver stiffness measurement; MASLD, metabolic dysfunction associated steatotic liver disease; MASH, metabolic dysfunction steatohepatitis.

Supplementary Table 2: Associations between continuous 1-year weight change (per 5%) with MASLD, at-risk MASH and increased LSM stratified for sex

	male			female		
	OR	95% CI	*P*	OR	95% CI	*P*
**MASLD**						
Weight gain	1.38	1.27 – 1.50	<0.001	1.17	1.10 – 1.24	< .001
Weight loss	0.66	0.60 – 0.72	<0.001	0.69	0.63 – 0.74	< .001
**At-risk MASH**						
Weight gain	1.20	1.10 – 1.31	<0.001	1.08	0.94 – 1.20	.247
Weight loss	0.81	0.70 – 0.93	0.003	0.79	0.66 – 0.93	.002
**LSM ≥ 8 kPa**						
Weight gain	1.22	1.12 – 1.33	<0.001	1.16	1.05 – 1.27	.002
Weight loss	0.84	0.73 – 0.94	0.005	0.76	0.66 – 0.86	< .001

Results were obtained with logistic regression models and given as OR with 95% CI for MASLD, at-risk MASH (based on FAST score ≥ 0.35) and LSM ≥ 8 kPa as outcome per 5% weight gain or 5% weight loss. The analysis included up to 3325 males and 3477 females. Results were adjusted in model 1 for age, ethnicity and weight (1 year ago). Abbreviations: CI, confidence interval; LSM, liver stiffness measurement; MASH, metabolic dysfunction associated steatohepatitis; MASLD, metabolic dysfunction associated steatotic liver disease; OR, odds ratio.

Supplementary Table 3: Associations between continuous 1-year weight change with MASLD, at-risk MASH and increased LSM for participants with ALT < 100 IU/L expressed per 5% weight change

	OR	95% CI	*P*
**MASLD**			
Weight gain	1.24	1.18 – 1.30	< .001
Weight loss	0.67	0.63 – 0.71	< .001
**At-risk MASH**			
Weight gain	1.14	1.06 – 1.23	< .001
Weight loss	0.78	0.70 – 0.88	< .001
**LSM ≥ 8 kPa**			
Weight gain	1.20	1.12 – 1.27	< .001
Weight loss	0.79	0.72 – 0.86	< .001

Results were obtained with logistic regression models and given as OR with 95% CI for MASLD, at-risk MASH (based on FAST score ≥ 0.35) and LSM ≥ 8 kPa as outcome per 5% weight gain or 5% weight loss. The analysis included up to 6753 individuals. Results were adjusted in model 1 for age, sex, ethnicity and prior weight (1 year). Abbreviations: CI, confidence interval; LSM, liver stiffness measurement; MASH, metabolic dysfunction associated steatohepatitis; MASLD, metabolic dysfunction associated steatotic liver disease; OR, odds ratio.

Supplementary Table 4: Associations between continuous 1-year weight change with MASLD, at-risk MASH and increased LSM for participants with daily ethanol intake < 20/30 gram in female and male expressed per 5% weight change

	OR	95% CI	*P*
**MASLD**			
Weight gain	1.24	1.18 – 1.30	< .001
Weight loss	0.67	0.63 – 0.71	< .001
**At-risk MASH**			
Weight gain	1.14	1.06 – 1.22	< .001
Weight loss	0.81	0.72 – 0.90	< .001
**LSM ≥ 8 kPa**			
Weight gain	1.19	1.12 – 1.27	< .001
Weight loss	0.79	0.72 – 0.87	< .001

Results were obtained with logistic regression models and given as OR with 95% CI for MASLD, at-risk MASH (based on FAST score ≥ 0.35) and LSM ≥ 8 kPa as outcome per 5% weight gain or 5% weight loss. The analysis included up to 6592 individuals. Results were adjusted in model 1 for age, sex, ethnicity and prior weight (1 year). Abbreviations: CI, confidence interval; LSM, liver stiffness measurement; MASH, metabolic dysfunction associated steatohepatitis; MASLD, metabolic dysfunction associated steatotic liver disease; OR, odds ratio.

Supplementary Table 5: Associations between continuous 1-year weight change with MASLD, at-risk MASH and increased LSM for participants trying to lose weight expressed per 5% weight change

	OR	95% CI	*P*
**MASLD**			
Weight gain	1.19	1.11 – 1.27	< .001
Weight loss	0.66	0.61 – 0.71	< .001
**At-risk MASH**			
Weight gain	1.10	0.99 – 1.21	.047
Weight loss	0.76	0.66 – 0.86	< .001
**LSM ≥ 8 kPa**			
Weight gain	1.23	1.13 – 1.33	< .001
Weight loss	0.72	0.64 – 0.81	< .001

Results were obtained with logistic regression models and given as OR with 95% CI for MASLD, at-risk MASH (based on FAST score ≥ 0.35) and LSM ≥ 8 kPa as outcome per 5% weight gain or 5% weight loss. The analysis included up to 3472 individuals. Results were adjusted in model 1 for age, sex, ethnicity and prior weight (1 year). Abbreviations: CI, confidence interval; LSM, liver stiffness measurement; MASH, metabolic dysfunction associated steatohepatitis; MASLD, metabolic dysfunction associated steatotic liver disease; OR, odds ratio.

Supplementary Figure 1: Weight loss and weight gain distribution

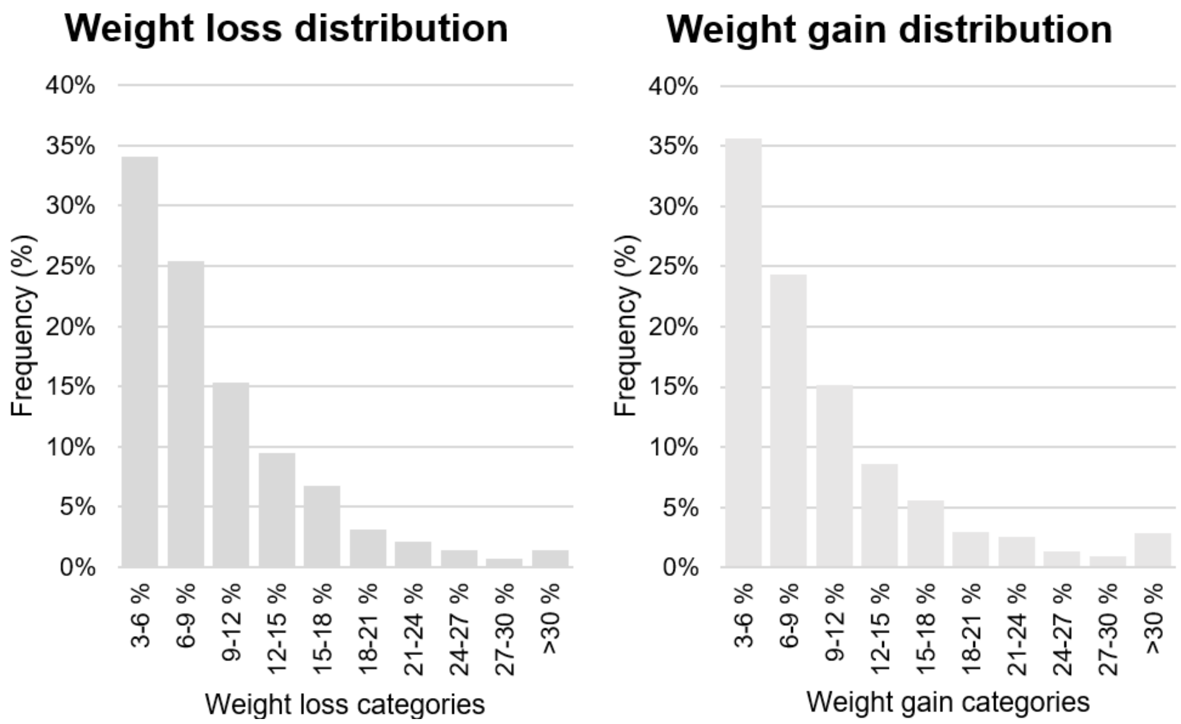

Weight loss of ≥ 3% was present in 1877 participants and weight gain of ≥ 3% in 1968.
