# Supplementary material for: Weight Change is Associated With Metabolic Liver Health in a General Population Extending Beyond Weight Loss Targets of International Guidelines
Source: Gastro Hep Adv. 2025 Oct 10;5(2):100831. doi: 10.1016/j.gastha.2025.100831 (PMC12670896; doi:10.1016/j.gastha.2025.100831)
Supplement: Extended PDF [file mmc2.pdf]

Gastro Hep Advances 2025;■:100831

ORIGINAL RESEARCH—CLINICAL

Weight Change is Associated With Metabolic Liver Health in a General Population Extending Beyond Weight Loss Targets of International Guidelines

Laurens A. van Kleef,[1] Mesut Savas,[2,3,*] Maurice Michel,[4,5,*] Cyrille Caussy,[6,7] Jesse Pustjens,[1] Adriaan G. Holleboom,[8,9] Elisabeth F. C. van Rossum,[2,3] Harry L. A. Janssen,[1,10] Jörn M. Schattenberg,[4,5] and Willem P. Brouwer[1,*]

[1]Department of Gastroenterology and Hepatology, Erasmus MC, University Medical Center, Rotterdam, The Netherlands; [2]Department of Internal Medicine, Division of Endocrinology, Erasmus MC, University Medical Center, Rotterdam, The Netherlands; [3]Obesity Center CGG, Erasmus MC, University Medical Center, Rotterdam, The Netherlands; [4]Department of Internal Medicine II, Saarland University Medical Center, Homburg, Germany; [5]Saarland University, Saarbrücken, Germany; [6]Hospices Civils de Lyon, Département Endocrinologie, Diabète et Nutrition, Hôpital Lyon Sud Pierre-Bénite, France; [7]Univ Lyon, CarMen Laboratory, INSERM, INRA, INSA Lyon, Université Claude Bernard Lyon 1 Pierre-Bénite, France; [8]Department of Vascular Medicine, Amsterdam UMC, The Netherlands; [9]Amsterdam Gastroenterology Endocrinology and Metabolism Institute, Amsterdam UMC, The Netherlands; and [10]Toronto Centre for Liver Disease, Toronto General Hospital, University Health Network, Canada

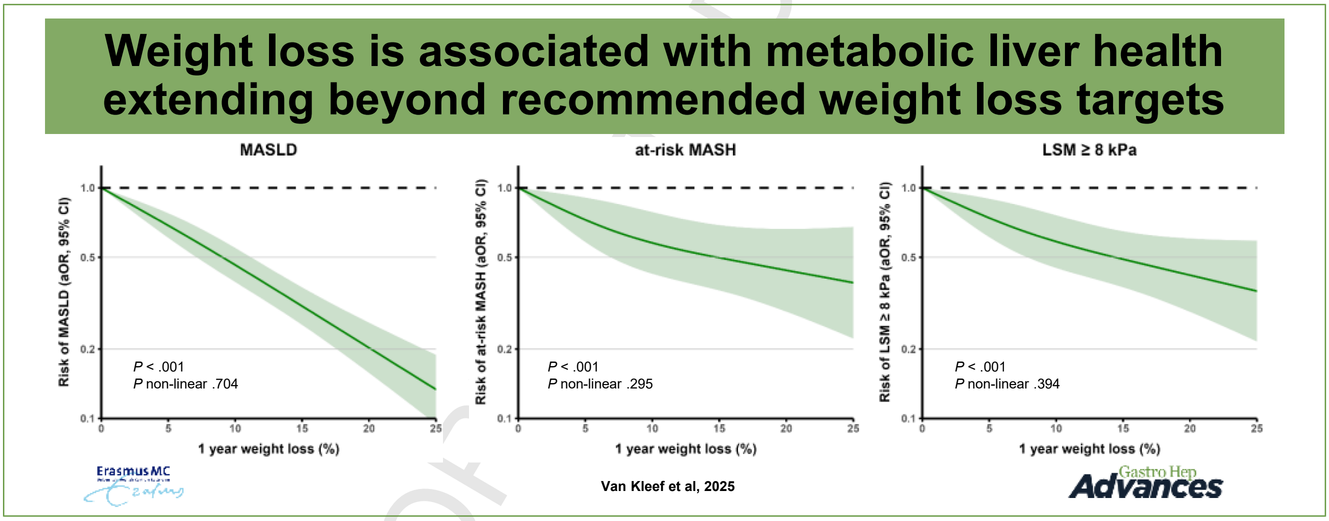

BACKGROUND AND AIMS: Weight loss of ≥3%–10% is recommended in metabolic dysfunction–associated steatotic liver disease (MASLD) management, according to current guidelines. We investigated the associations between weight change and impaired metabolic liver health and focused on associations beyond these recommendations. **METHODS:** Adults from the National Health and Nutrition Examination Survey 2017–2020, with data on 1-year weight history, controlled attenuation parameter and/or liver stiffness measurement (LSM) were selected. Exclusion criteria were age ≥80 years, body mass index <18.5 kg/m^2, excessive alcohol and viral hepatitis. Impaired metabolic liver health included MASLD (controlled attenuation parameter ≥275 dB/m with ≥1 cardiometabolic riskfactor), at-risk metabolic dysfunction–associated steatohepatitis (MASH) (FibroScan aspartate aminotransferase score ≥0.35) and LSM ≥8 kPa. Multivariate logistic regression models were adjusted for demographics and prior weight. **RESULTS:** We included 6802 individuals (aged 48 years [33–62], 48.9% male). MASLD was present in 42.2%, at-risk MASH in 6.5% and LSM ≥8 kPa in 9.1%. Over 1 year, 29% gained and 28% lost

*Indicates shared authorship

Abbreviations used in this paper: **aOR, adjusted Odds Ratio; BMI, Body Mass Index; CAP, Controlled Attenuation Parameter; CI, Confidence Interval; LSM, Liver Stiffness Measurement; MASLD, Metabolic dysfunction-associated steatotic liver disease; MASH, Metabolic dysfunction-associated steatohepatitis; NHANES, National Health and Nutrition Examination Survey; VCTE, Vibration Controlled Transient Elastography.**

Copyright © 2025 The Authors. Published by Elsevier Inc. on behalf of the AGA Institute. This is an open access article under the CC BY license (http://creativecommons.org/licenses/by/4.0/).
2772-5723
https://doi.org/10.1016/j.gastha.2025.100831

≥3% weight. Compared to stable weight, weight gain ≥3% was associated with increased MASLD prevalence (adjusted odds ratio (aOR):1.78; 95% confidence interval (CI): 1.48–1.95), at-risk MASH (aOR: 1.78; 95% CI: 1.39–2.29) and LSM ≥8 kPa (aOR:1.48; 95%CI:1.19–1.84); whilst weight loss ≥ 3% was associated with reduced MASLD prevalence (aOR: 0.54; 95% CI: 0.47–0.62), at-risk MASH (aOR: 0.72; 95% CI: 0.55–0.94) and LSM ≥8 kPa (aOR: 0.62; 95% CI: 0.49–0.78). Results were consistent when weight loss was further categorized or when assessed as continuous variable without evidence for nonlinearity. **CONCLUSION:** The prevalence of impaired metabolic liver health decreased with weight loss. Greater reported weight loss was associated with lower observed risks. Hence, we should recommend losing weight beyond the currently recommended targets to further reduce the risk of advanced liver disease.

Keywords: Liver Health; MASLD; MASH; Fibrosis; Liver Stiffness; General Population; Obesity; Weight Change; Weight Loss; Epidemiology

Introduction

Recent findings suggest that the method of weight loss—whether through lifestyle intervention, incretin-based therapies, bariatric surgery, or a combination—does not influence the improvement of metabolic dysfunction–associated steatotic liver disease (MASLD).[1,2] This emerging paradigm, supported by the interim analysis of the Essence III trial, positions weight loss as the key driver of MASLD regression.[3]

Indeed, the European clinical practice guidelines recommend weight loss ranging from 3%–5% among those with normal weight while ≥5% is recommended for individuals with overweight or obesity and MASLD, increasing to ≥7%–10% when steatohepatitis or fibrosis is present.[4] The American Association for the Study of Liver Diseases practice guidance mentions 3%–5% weight loss for improvement in steatosis but notes that greater weight loss (>10%) might be needed for steatohepatitis and fibrosis.[5] The American Gastroenterological Association clinical care pathway recommends 5% weight loss for steatosis and up to 10% for steatohepatitis and fibrosis.[6] Moreover, all of these guidelines recommend considering incretin-based therapy or bariatric surgery when weight loss targets are not obtained through lifestyle modifications.[4–6]

Although there is no doubt about the effectiveness of weight loss on the improvements across the MASLD spectrum, there is no consensus on the optimal weight loss target, illustrated by the differences among the guidelines. Current targets are primarily based on a study of 293 MASLD patients who underwent liver biopsy, of which 61% had ≥ F1 fibrosis in biopsy.[7] They demonstrated that there was no incident fibrosis among the 48 individuals that had ≥5% weight loss whereas 27% had incident fibrosis when <5% weight loss was obtained. Similarly, no worsening of fibrosis was observed in 24 individuals with ≥7% weight loss, whereas up to 81% showed improvement.[7] Other cited studies in the guidelines include primarily meta-analyses of controlled trials that either compare the efficacy of weight loss interventions (and not weight loss itself) or focus on steatosis and not on liver stiffness or liver fibrosis due to limited data.[8–10]

To strengthen the evidence for weight loss in MASLD we therefore investigated the association between weight loss and weight gain with MASLD, at-risk metabolic dysfunction–associated steatohepatitis (MASH) and increased liver stiffness in a population-based study reflecting a real-world setting.

Methods

Study Population

Study participants were selected from the National Health and Nutrition Examination Survey (NHANES) 2017–2020. The aim of NHANES is to assess the health and nutritional status among a United States representative population.[11] For this purpose, they prospectively collect data by extensive interviews, physical examination, clinical measurements and blood tests by dedicated research assistants. In this study, we primarily used data on the weight history questionnaire as well as liver health assessment by vibration-controlled transient elastography (VCTE) using FibroScan. Adult participants were eligible for inclusion when they had complete data on weight history, liver stiffness measurement (LSM) and controlled attenuation parameter (CAP) by VCTE. Exclusion criteria were age ≥ 80 years, currently being underweight defined as body mass index (BMI) ≤ 18.5 kg/m^2, ≥ 60 g of alcohol on a daily basis and presence of hepatitis B or hepatitis C. Data are publicly available from the NHANES database (https://www.cdc.gov/nchs/nhanes/index.htm).

Weight History

The weight history questionnaire included data on current and prior (1 year and 10 years) weight. Absolute weight change (kg) was based on the difference between self-reported current weight and prior weight for both 1-year weight loss and 10-year weight loss. Previous data indicate that self-reported weight correlates very well with actual measured weight and can, therefore, be safely used as an exposure variable in this study.[12] Additionally, percentage weight loss was calculated based on dividing the absolute weight change by the prior weight. Based on the weight loss recommendations in the guidelines we defined, weight gain as an increase equal to or more than 3% body weight, and weight loss as a decrease equal to or more than 3% body weight. Stable weight was considered when reported weight change was between −3 and +3% of body weight.[4–6] The weight history questionnaire additionally includes data on whether individuals attempted losing weight last year.

Liver Outcomes

VCTE was performed using the FibroScan model 502 V2 Touch (FibroScan, Echosens, Paris) in participants who were instructed to fast for ≥ 3 hours. With VCTE the LSM and CAP were assessed. Measurements were performed with either the M-probe or XL-probe, based on the instructions of the device. VCTE readings were subsequently used to define the following conditions.

UNCORRECTED PROOF

ARTICLE IN PRESS

- MASLD: CAP ≥ 275 dB/m together with ≥1 cardiometabolic risk factor[13,14]
- At-risk MASH: FibroScan aspartate aminotransferase (FAST) score ≥ 0.35[15]
- Increased LSM: LSM ≥ 8 kPa in the absence of heart failure, indicating high risk of ≥ F2 fibrosis[16,17]

Collectively these outcomes are referred to as “impaired metabolic liver health”.[18]

Covariates

This study further used questionnaire data (*eg* medical history for heart failure, diabetes, hypertension and list of prescribed medications), blood samples (*eg* liver enzymes, fasting glucose, hemoglobin A1c and lipids).

Cardiometabolic risk factors were scored according to the definitions for the diagnosis of MASLD:[14]

(1) BMI ≥ 25 kg/m^2 (or ≥23 kg/m^2 in Asians) or waist circumference ≥ 94 cm in male and ≥80 cm in female;
(2) T2DM or fasting glucose ≥ 5.6 mmol/L or hemoglobin A1c ≥ 5.7%; Q5
(3) Blood pressure ≥ 130/85 mmHg or antihypertensive drug treatment;
(4) Triglycerides ≥ 1.7 mmol/L or lipid-lowering treatment;
(5) Hypodensity lipoprotein ≤ 1.0 mmol/L in males or ≤ 1.3 mmol/L in females or lipid-lowering treatment.

Statistical Analysis

We used logistic regression analysis to quantify the associations between weight change and MASLD, at-risk MASH and LSM ≥8 kPa. Weight change was first evaluated categorically. Multivariate logistic models were adjusted for covariates for demographics (age, sex, and race) and prior weight (model 1). Additionally, in model 2, we adjusted for stable socioeconomic factors: education, smoking and alcohol consumption.[19] Next, we focused on percentage weight change on a continuous scale, using the same multivariate models. To investigate whether there are ongoing effects beyond the current guidelines, restricted cubic splines analysis was used to assess nonlinearity between weight change and the outcomes. Based on the restricted cubic splines analysis, the weight change variable was separated into percentage weight gain and percentage weight loss on a continuous scale. Extending on this, analyses were stratified according to prior weight status. In sensitivity analyses using the continuous data, we (1) replaced 1-year weight with 10-year weight change; (2) analysis stratified by sex; (3) excluded individuals with alanine aminotransferase ≥ 100 IU/L; and (4) excluded individuals with alcohol consumption ≥ 20 g per day for female and ≥ 30 g per day for male. Analyses were performed in R version 4.0.4 (Foundation for Statistical Computing, Vienna, Austria). *P* values < 0.05 were considered statistically significant.

Results

Participant Characteristics

For this study, 7.581 adult participants from NHANES with data on LSM, CAP, and weight history were eligible for inclusion. Of these, 409 were excluded for being aged ≥ 80 years, 171 for being underweight, 95 for excessive alcohol consumption and 104 for viral hepatitis, leaving 6.802 participants for analysis. The median age of study participants was 48 [33–62] years; 48.9% were male; 1-year prior BMI was 28.6 kg/m^2 [24.8–33.6] and 51.0% attempted losing weight (of which 293 [8.4%] on pharmaceutical treatment, 21 [0.6%] bariatric surgery). In the year prior to the study visit, 43.5% had no substantial weight change, 27.6% had weight loss (≥3% body weight loss) and 28.9% had weight gain (≥3% body weight gain). In the 10 years prior to the study visit, 18.1% reported stable weight, 26.7% weight loss and 55.2% weight gain. Among those with weight loss over the past year, the median weight loss was 7.7% [5.2%–12.0%], similar to the weight gain among those with weight gain (median 7.6% [5.1%–11.9%]). The distribution of weight change is further visualized in Figure A1. At the study visit, 42.2% had MASLD, 6.5% at-risk MASH and 9.1% increased LSM. Additional characteristics can be found in Table 1 and characteristics after stratification for weight change status in Table A1.

Table 1. Participant Characteristics

n	6.802
Demographics	
Age	48 [33, 62]
Male	3325 (48.9)
Ethnicity	
Asian	866 (12.7)
Black	1805 (26.5)
Hispanic	1571 (23.1)
Other	351 (5.2)
White	2209 (32.5)
Comorbidity	
BMI kg/m^2 (1-y prior)	28.6 [24.8, 33.6]
Weight status (1-y prior)	
BMI ≥ 30 kg/m^2	2563 (38.3)
BMI 25–30 kg/m^2	2088 (31.2)
BMI < 25 kg/m^2	2036 (30.4)
Diabetes	1220 (18.6)
Hypertension	3198 (49.9)
High waist circumference	3866 (58.2)
Biochemistry	
AST	19 [16, 23]
ALT	18 [13, 26]
HDL	1.4 (0.4)
Triglycerides	1.3 [0.9, 1.8]
Outcomes	
MASLD	2870 (42.2)
At-risk MASH	414 (6.5)
LSM ≥ 8 kPa	607 (9.1)
CAP	264 (62)
LSM	5.0 [4.1, 6.1]

Data are presented as mean (SD), median [P25-P75] or n and percentage.
ALT, alanine aminotransferase; AST, aspartate aminotransferase; HDL, hypodensity lipoprotein.

UNCORRECTED PROOF

Associations Between Categorical Weight Change and Metabolic Liver Health

Weight gain ≥3% over the past year was associated with increased prevalence of MASLD (aOR 1.70, 95% CI 1.48–1.95), at-risk MASH (aOR 1.78, 95% CI 1.39–2.29) and LSM ≥ 8 kPa (aOR 1.48, 95% CI 1.19–1.84) Table 2. On the other hand, weight loss ≥3% over the past year resulted in lower prevalence of MASLD (aOR 0.54, 95% CI 0.47–0.63), at-risk MASH (aOR 0.69, 95% CI 0.52–0.92) and LSM ≥ 8 kPa (aOR 0.61, 95% CI 0.48–0.77). Further adjusting for socioeconomic status and substance use (education level, current smoking status, and alcohol consumption) yielded similar effect estimates. However, the association between weight loss and at-risk MASH was no longer significant (aOR 0.77, 95% CI 0.58–1.02). Additional categorization of weight change indicated stronger associations for weight loss ≥ 15% body weight compared to 3%–10% or 10%–15% for all outcomes Table 3.

Associations Between Continuous Weight Change and Metabolic Liver Health

On a continuous scale, 1-year weight gain was associated with increased prevalence of MASLD (aOR 1.24 per 5%, 95% CI 1.19–1.31) at-risk MASH (aOR 1.15 per 5%, 95% CI 1.07–1.23) and LSM ≥ 8 kPa (aOR 1.19 per 5%, 95% CI 1.12–1.26). Similarly, each 5% of weight loss for the associations with MASLD (aOR 0.67 per 5%, 95% CI 0.62–0.71), at-risk MASH (aOR 0.82 per 5%, 95% CI 0.73–0.91) and LSM ≥ 8 kPa (aOR 0.79 per 5%, 95% CI 0.71–0.86). Results were consistent after further adjusting for socioeconomic factors or when focusing on 10-year weight change; Table 4. For weight loss, there was no evidence for nonlinearity, indicating ongoing effects with more weight loss, Figure 1. In contrast with weight gain, which was associated with MASLD and at-risk MASH in a nonlinear fashion: associations attenuated or even plateaued after 10% weight gain (Figure 2).

Impact of BMI Category on the Associations Between Weight Change and Metabolic Liver Health

Next, we focused on the potential effects of the baseline BMI category (18.5–25, 25–30, and ≥ 30 kg/m^2) on the observed associations. Here we demonstrate consistent effects among individuals living with obesity or with overweight when compared to the unstratified population. However, among participants reporting normal weight 1 year prior to the current visit, the associations attenuated (Table 5).

Sensitivity Analysis

Results were consistent when stratified for sex (Table A2), after excluding individuals with alanine aminotransferase > 100 IU/L (Table A3), after excluding individuals with alcohol consumption between 20/30 and 60 g of alcohol per day in female and male (Table A4) and after focusing on individuals that aimed for weight loss. (Table A5).

Discussion

Weight loss is an important pillar in MASLD management, with recommendations varying between ≥3%–10%; however, data are scarce with the most recent meta-analysis only including 2809 individuals, and evidence is not available other than from a secondary care perspective.[4–6,9] We investigated the associations between impaired metabolic liver health with self-reported weight change in a population based setting and demonstrated that the prevalence of impaired metabolic liver health was

Table 2. Associations Between Categorical 1-Year Weight Change With MASLD, At-Risk MASH and Increased LSM

			Model 1			Model 2		
	N	n	OR	95% CI	*P*	OR	95% CI	*P*
MASLD								
Stable weight	2957	1246		Reference			Reference	
Weight gain	1968	850	1.70	1.48–1.95	<.001	1.73	1.50–2.00	<.001
Weight loss	1877	774	0.54	0.47–0.62	<.001	0.54	0.47–0.63	<.001
At-risk MASH								
Stable weight	2742	162		Reference			Reference	
Weight gain	1836	140	1.78	1.39–2.29	<.001	1.95	1.50–2.54	<.001
Weight loss	1748	112	0.72	0.55–0.94	.016	0.77	0.58–1.02	.069
LSM ≥ 8 kPa								
Stable weight	2894	262		Reference			Reference	
Weight gain	1935	179	1.48	1.19–1.84	<.001	1.53	1.22–1.91	<.001
Weight loss	1811	166	0.62	0.49–0.78	<.001	0.62	0.49–0.78	<.001

Results were obtained with logistic regression models and given as OR, with 95% CI for MASLD, at-risk MASH (based on FAST, score ≥ 0.35) and LSM ≥ 8 kPa as outcome. Results were adjusted in model 1 for age, sex, ethnicity, and prior weight (1 year ago); model 2 in addition for education, alcohol, and smoking.
N, total number in subgroup, n, number of cases.

Table 3. Risk of MASLD, At-Risk MASH and Increased LSM for Further Categorized Weight Change Categories

	MASLD		At-risk MASH		LSM ≥ 8 kPa	
	OR	95% CI	OR	95% CI	OR	95% CI
Weight change						
<−15%	0.19	0.14–0.26	0.48	0.27–0.82	0.42	0.26–0.66
−15% to −10%	0.44	0.34–0.58	0.71	0.43–1.13	0.69	0.46–1.02
−10% to −3%	0.68	0.58–0.80	0.80	0.59–1.07	0.66	0.51–0.85
−3 to + 3%	Reference		Reference		Reference	
+ 3 to + 10%	1.52	1.30–1.77	1.73	1.31–2.28	1.31	1.03–1.68
+ 10 to + 15%	1.77	1.36–2.30	1.44	0.84–2.34	1.74	1.14–2.58
> + 15%	2.80	2.13–3.69	2.67	1.63–4.23	2.29	1.46–3.49

Table 4. Associations Between Continuous Weight Change With MASLD, At-Risk MASH and Increased LSM Expressed per 5% Weight Change Q9

		Model 1			Model 2		
		OR	95% CI	*P*	OR	95% CI	*P*
MASLD							
Weight gain	1 y	1.24	1.19–1.31	<.001	1.25	1.19–1.32	<.001
Weight loss	1 y	0.67	0.63–0.71	<.001	0.67	0.62–0.71	<.001
Weight gain	10 y	1.30	1.26–1.34	<.001	1.30	1.26–1.35	<.001
Weight loss	10 y	0.69	0.65–0.73	<.001	0.68	0.64–0.72	<.001
At-risk MASH							
Weight gain	1 y	1.15	1.07–1.23	<.001	1.16	1.08–1.25	<.001
Weight loss	1 y	0.81	0.72–0.89	<.001	0.82	0.73–0.91	<.001
Weight gain	10 y	1.13	1.09–1.18	<.001	1.14	1.09–1.19	<.001
Weight loss	10 y	0.77	0.69–0.85	<.001	0.78	0.70–0.86	<.001
LSM ≥ 8 kPa							
Weight gain	1 y	1.19	1.12–1.26	<.001	1.18	1.11–1.26	<.001
Weight loss	1 y	0.80	0.72–0.87	<.001	0.79	0.71–0.86	<.001
Weight gain	10 y	1.16	1.12–1.21	<.001	1.17	1.13–1.22	<.001
Weight loss	10 y	0.80	0.74–0.87	<.001	0.80	0.73–0.87	<.001

Results were obtained with logistic regression models and given as OR, with 95% CI, for MASLD, at-risk MASH (based on FAST, score ≥ 0.35) and LSM ≥ 8 kPa as outcome per 5% weight gain or 5% weight loss. The 1-year analysis included up to 6802 participants and the 10-years analysis 4722 participants. Results were adjusted in model 1 for age, sex, ethnicity and prior weight (either 1 year or 10 years ago).

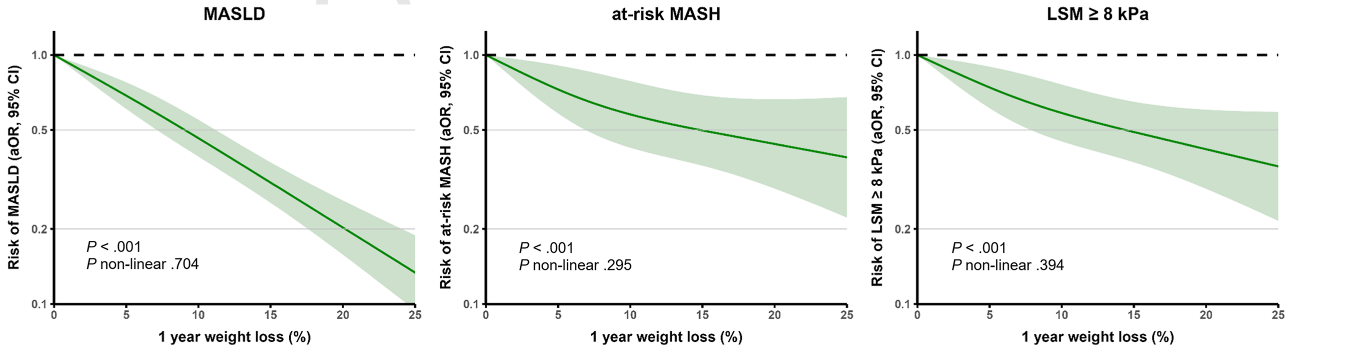

Figure 1. Association between 1-year weight loss and impaired liver health—assessment for nonlinearity. Restricted cubic spline analysis was performed using 3 knots. Results were adjusted for age, sex, ethnicity, and prior weight.

CORRECTED PROOF

ARTICLE IN PRESS

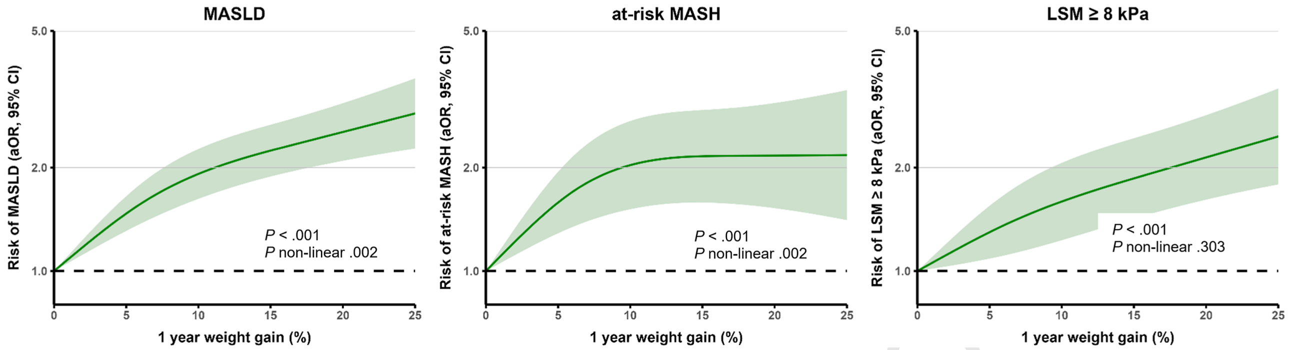

Figure 2. Association between 1-year weight gain and impaired liver health—assessment for nonlinearity.

associated with weight change history. Importantly, more weight loss was associated with larger effect sizes for risk of MASLD, at-risk MASH and LSM $\geq$ 8 kPa without evidence for nonlinearity; implicating increasing risk reductions for weight loss extending beyond current guideline recommendations. Although ultimately longitudinal confirmation of these findings is required, our results provide new population-based evidence suggesting that greater weight loss may be beneficial in MASLD and its complications, particularly among individuals who remain overweight or obese after achieving a $\geq$3%–10% weight reduction.

The observed associations are particularly important for individuals living with obesity, who account for the majority of at-risk MASH and increased LSM cases. Weight loss recommendations need to be tailored to the current weight class (overweight, obesity, etc), based on stronger associations for all investigated outcomes in individuals with obesity compared to participants without obesity. In fact, among participants with BMI $<$ 25 kg/m^2 we could only demonstrate a significant risk reduction between weight loss and lower prevalence of MASLD whereas no significant associations were demonstrated with at-risk MASH and increased LSM. Although this could be lack of statistical power, this also indicates that individuals with normal weight may require other interventions and weight loss might not be as beneficial when compared to individuals with overweight/obesity, despite currently being an important target in lean-MASLD.[20]

The hepatoprotective associations of weight loss can be attributed to an overall improvement of the cardiometabolic risk profile previously demonstrated in a population-based setting.[21] Similar to our findings, increasingly positive effects of weight-loss were demonstrated across the entire observed weight loss spectrum. These findings align with a recent evaluation of a bariatric surgery population where the regression of MASH increased to 20% but plateaued between 20% and 25% weight loss; which in our study population was not reached frequently.[1] This further supports moving away from weight loss targets and focus disease regression which may require more weight loss then currently recommended.[22] Particularly relevant for these associations is the decrease of visceral adipose tissue, a key

Table 5. Associations Between Continuous 1-Year Weight Change With MASLD, At-Risk MASH, and Increased LSM Expressed per 5% Weight Change According to Weight Status

	Weight gain					Weight loss				
	N	n	OR	95% CI	*P*	N	n	OR	95% CI	*P*
MASLD										
BMI $\geq$ 30 kg/m^2	553	392	1.40	1.26–1.56	<.001	1081	608	0.73	0.68–0.78	<.001
BMI 25–30 kg/m^2	602	299	1.33	1.23–1.45	<.001	505	127	0.58	0.50–0.67	<.001
BMI $<$ 25 kg/m^2	776	142	1.12	1.05–1.19	.001	262	23	0.42	0.29–0.57	<.001
At-risk MASH										
BMI $\geq$ 30 kg/m^2	515	68	1.22	1.07–1.38	.002	1014	93	0.84	0.75–0.94	.003
BMI 25–30 kg/m^2	562	41	1.25	1.10–1.40	<.001	467	14	0.69	0.47–0.95	.042
BMI $<$ 25 kg/m^2	726	28	1.09	0.94–1.21	.160	240	3	0.71	0.32–1.20	.288
LSM $\geq$ 8 kPa										
BMI $\geq$ 30 kg/m^2	542	113	1.36	1.23–1.51	<.001	1032	128	0.78	0.70–0.86	<.001
BMI 25–30 kg/m^2	586	40	1.18	1.03–1.34	.010	498	22	0.82	0.61–1.04	.127
BMI $<$ 25 kg/m^2	770	22	1.10	0.97–1.22	.081	253	14	1.53	1.13–1.99	.003

Results were obtained with logistic regression models and given as OR, with 95% CI, for MASLD, at-risk MASH (based on FAST, score $\geq$ 0.35) and LSM $\geq$ 8 kPa as outcome per 5% weight gain or 5% weight loss. Number and events for $\geq$ 3% weight gain or weight loss are reported. Of note, participants with stable weight are included in the analyses but not included in the reported numbers and events. Results were adjusted for age, sex, ethnicity and prior weight (1 year ago).

FLA 5.7.0 DTD ■ GASTHA100831_proof ■ 27 October 2025 ■ ce

driver of MASLD but also important in the progression from steatosis to steatohepatitis and fibrosis.[23] In this study, muscle mass loss (*eg* in light of sarcopenia) could not be differentiated from visceral adipose tissue, and the effects of pure visceral adipose tissue loss are likely to exceed the reported associations.[24]

Although weight loss is difficult to achieve and even harder to maintain, this study shows increasing benefits along the entire weight loss spectrum.[25] Therefore, the targeted weight loss in patients with MASLD should not be a static number but a dynamic process that can adapt during disease evaluation and take into account the current weight status. Additional follow-up with hepatic assessment is important when the initial weight goal has been obtained and recommending further obesity treatment should be considered when there is no disease regression.

We demonstrated that weight gain was associated with an increased prevalence of MASLD, at-risk MASH or LSM ≥8 kPa in a real-world setting among those with overweight and obesity. The observational time frame might have been too short to demonstrate this for normal weight individuals as well, as it takes several years to develop MASH or fibrosis.[23] This indicates that weight (re)gain should be prevented, particularly among individuals at-risk of MASLD and its complications. Previous data from patients living with HIV support these findings and demonstrate that a ≥5% BMI increase was associated with a threefold risk of fibrosis onset or progression based on VCTE.[26] Moreover, in this cohort using the FibroScan aspartate aminotransferase score for at-risk MASH and liver stiffness we confirm findings from a Korean population-based study, which demonstrated that 20% of the population gaining the most weight were at 1.7 times higher risk of fibrosis progression based on APRI during a median follow-up of 7 years.[27]

The current study adds to the evidence that risk reduction can even be observed in a very short period of time since the effect size of a 1-year change was not substantially different from that of a 10-year change in the continuous analysis. This aligns with current ongoing trials investigating novel compounds for treatment of fibrotic MASH which already show improvements after a few months.[28] The current findings are reassuring, as they indicate that the risk attributed to overweight and obesity is reversible based on this population-based study, and the so-called metabolic memory might have a limited impact on the risk of liver disease when weight loss is obtained and maintained.[29] These findings align with recent insights that the risk for impaired metabolic liver health attributed to young adulthood obesity is largely reversible when weight loss is obtained later in life.[30]

Although this study assessed the role of weight loss in prevalence of MASLD, at-risk MASH and LSM ≥8 kPa in more than 6.000 individuals in a real-world setting, the following limitations should be noted. First, reliability of liver stiffness readings among individuals with obesity is debated.[31] However, a recent meta-analysis of over 10.000 patients showed that LSM was reliable when using the XL probe. CAP levels, on the other hand, might indeed be falsely elevated in individuals with obesity.[32] Correlating these findings with histology or with magnetic resonance imagining proton density fat fraction would be particularly interesting but it is not feasible in this population-based setting. Second, this study could not clearly differentiate between how weight loss was obtained and no meaningful analyses could be performed on whether weight loss obtained through (moderated) lifestyle intervention, pharmaceutical treatment or surgery was equally effective. Moreover, there was also weight loss in individuals who already had normal weight, which may introduce bias due to reversed causality (*eg* weight loss due to disease progression which could be due to other causes than MASLD).[33] Third, data on impaired metabolic liver health were only available at a single time point, therefore we could not assess longitudinal changes, such as progression or regression of liver disease. Consequently, it was not possible to examine temporal trends or causal relationships. Fourth, data on prior weight were self-reported, potentially leading to differences with actual prior weight; however, previous data obtained in US individuals indicate that self-reported weight is highly accurate and can safely be used for analyses.[12] Aligning with this, no data were available on other markers of obesity such as waist circumference which together with body weight better reflects disease mechanisms and liver disease involvement.[34,35] Fifth, ALT > 100 IU/L might falsely elevate liver stiffness (although unlikely to be directly caused by weight change) it could obscure the reported findings. However, similar results were obtained in sensitivity analysis when these individuals (n = 49) were excluded. Finally, although NHANES is designed to reflect a general population, population-based studies are affected by response rates, which may introduce selection bias and limit the extent to which the study sample truly represents the broader population.

Conclusion

In this general population study the amount of weight loss required for optimal risk reduction might exceed the current guideline recommendations. The prevalence of MASLD, at-risk MASH and LSM ≥8 kPa was strongly increased with weight gain and reduced with weight loss. Importantly, more weight loss was associated with stronger reduced risk, without evidence for attenuation of these effects. This indicates that weight gain should be prevented, and weight loss should be considered if MASLD is present to reduce the risk of advanced liver disease - even when initial weight loss targets have been reached.

Supplementary Materials

Material associated with this article can be found, in the online version, at https://doi.org/10.1016/j.gastha.2025.100831.

UNCORRECTED PROOF

References

1. Verrastro O, Panunzi S, Castagneto-Gissey L, et al. Bariatric–metabolic surgery versus lifestyle intervention plus best medical care in non-alcoholic steatohepatitis (BRAVES): a multicentre, open-label, randomised trial. Lancet 2023;401:1786–1797.
2. Machado MV. MASLD treatment-a shift in the paradigm is imminent. Front Med (Lausanne) 2023;10:1316284.
3. Newsome PN, Sanyal AJ, Engebretsen KA, et al. Semaglutide 2.4 mg in participants with metabolic dysfunction-associated steatohepatitis: baseline characteristics and design of the phase 3 ESSENCE trial. Aliment Pharmacol Ther 2024.
4. Tacke F, Horn P, Wai-Sun Wong V, et al. EASL–EASD–EASO clinical practice guidelines on the management of metabolic dysfunction-associated steatotic liver disease (MASLD). J Hepatol 2024;81(3):492–542.
5. Rinella ME, Neuschwander-Tetri BA, Siddiqui MS, et al. AASLD practice guidance on the clinical assessment and management of nonalcoholic fatty liver disease. Hepatology 2023;77(5):1797–1835.
6. Kanwal F, Shubrook JH, Adams LA, et al. Clinical care pathway for the risk stratification and management of patients with nonalcoholic fatty liver disease. Gastroenterology 2021;161:1657–1669.
7. Vilar-Gomez E, Martinez-Perez Y, Calzadilla-Bertot L, et al. Weight loss through lifestyle modification significantly reduces features of nonalcoholic steatohepatitis. Gastroenterology 2015;149:367–378.e5.
8. Fernandez T, Vinuela M, Vidal C, et al. Lifestyle changes in patients with non-alcoholic fatty liver disease: a systematic review and meta-analysis. PloS One 2022;17:e0263931.
9. Koutoukidis DA, Koshiaris C, Henry JA, et al. The effect of the magnitude of weight loss on non-alcoholic fatty liver disease: a systematic review and meta-analysis. Metabolism 2021;115:154455.
10. Koutoukidis DA, Astbury NM, Tudor KE, et al. Association of weight loss interventions with changes in biomarkers of nonalcoholic fatty liver disease: a systematic review and meta-analysis. JAMA Intern Medicine 2019;179:1262–1271.
11. Johnson CL, Paulose-Ram R, Ogden CL, et al. National health and nutrition examination survey. Analytic guidelines, 1999-2010. Vital Health Stat 2013;1–24.
12. Hodge JM, Shah R, McCullough ML, et al. Validation of self-reported height and weight in a large, nationwide cohort of US adults. PloS One 2020;15:e0231229.
13. EASL clinical practice guidelines on non-invasive tests for evaluation of liver disease severity and prognosis – 2021 update. J Hepatol 2021;75:659–689.
14. Rinella ME, Lazarus JV, Ratziu V, et al. A multisociety Delphi consensus statement on new fatty liver disease nomenclature. J Hepatol 2023;79:1542–1556.
15. Newsome PN, Sasso M, Deeks JJ, et al. FibroScan-AST (FAST) score for the non-invasive identification of patients with non-alcoholic steatohepatitis with significant activity and fibrosis: a prospective derivation and global validation study. Lancet Gastroenterol Hepatol 2020;5:362–373.
16. Roulot D, Costes JL, Buyck JF, et al. Transient elastography as a screening tool for liver fibrosis and cirrhosis in a community-based population aged over 45 years. Gut 2011;60:977–984.
17. van Kleef LA, Sonneveld MJ, Zhu F, et al. Liver stiffness is associated with excess mortality in the general population driven by heart failure: the Rotterdam study. Liver Int 2023;43:1000–1007.
18. Ginès P, Guha IN, Fabrellas N, et al. Liver health: an emerging concept. Gastroenterology 2025;169:4–8.e1.
19. Kawado M, Suzuki S, Hashimoto S, et al. Smoking and drinking habits five years after baseline in the JACC study. J Epidemiol 2005;15(Suppl 1):S56–S66.
20. Long MT, Noureddin M, Lim JK. AGA clinical practice update: diagnosis and management of nonalcoholic fatty liver disease in lean individuals: expert review. Gastroenterology 2022;163:764–774.
21. Knell G, Li Q, Pettee Gabriel K, et al. Long-Term weight loss and metabolic health in adults concerned with maintaining or losing weight: findings from NHANES. Mayo Clin Proc 2018;93:1611–1616.
22. Sherifali D, Racey M, Fitzpatrick-Lewis D, et al. Missing the target: a scoping review of the use of percent weight loss for obesity management. Obes Rev 2025;26:e13960.
23. Israelsen M, Francque S, Tsochatzis EA, et al. Steatotic liver disease. Lancet 2024;404:1761–1778.
24. Alferink LJM, Trajanoska K, Erler NS, et al. Nonalcoholic fatty liver disease in the Rotterdam study: about muscle mass, sarcopenia, fat mass, and fat distribution. J Bone Mineral Research 2019;34:1254–1263.
25. Hall KD, Kahan S. Maintenance of lost weight and long-term management of obesity. Med Clin North Am 2018;102:183–197.
26. Guaraldi G, Milic J, Renzetti S, et al. The effect of weight gain and metabolic dysfunction-associated steatotic liver disease on liver fibrosis progression and regression in people with HIV. AIDS 2024;38:1323–1332.
27. Kim Y, Chang Y, Cho YK, et al. Obesity and weight gain are associated with progression of fibrosis in patients with nonalcoholic Fatty liver disease. Clin Gastroenterol Hepatol 2019;17:543–550.e2.
28. Harrison SA, Taub R, Neff GW, et al. Resmetirom for nonalcoholic fatty liver disease: a randomized, double-blind, placebo-controlled phase 3 trial. Nat Medicine 2023;29:2919–2928.
29. Dong H, Sun Y, Nie L, et al. Metabolic memory: mechanisms and diseases. Signal Transduct Target Ther 2024;9:38.
30. van Kleef LA, Pustjens J, Savas M, et al. MASLD, at-risk MASH and increased liver stiffness are associated with young adulthood obesity without residual risk after losing obesity. Liver Int 2025;45:e16169.
31. Chouik Y, Aubin A, Maynard-Muet M, et al. The grade of obesity affects the noninvasive diagnosis of advanced fibrosis in individuals with MASLD. Obesity (Silver Spring) 2024;32:1114–1124.
32. Cao Y-T, Xiang L-L, Qi F, et al. Accuracy of controlled attenuation parameter (CAP) and liver stiffness measurement (LSM) for assessing steatosis and fibrosis in non-alcoholic fatty liver disease: a systematic review and meta-analysis. EClinicalMedicine 2022;51:101547.

UNCORRECTED PROOF

ARTICLE IN PRESS

33. Sattar N, Preiss D. Reverse causality in cardiovascular epidemiological research: more common than imagined? Am Heart Assoc 2017;135:2369–2372.
34. Busetto L, Dicker D, Frühbeck G, et al. A new framework for the diagnosis, staging and management of obesity in adults. Nat Med 2024;30:2395–2399.
35. van Kleef LA, Michel M, Savas M, et al. A comparison of the predictive value of 12 body composition markers for metabolic dysfunction-associated steatotic liver disease, at-risk metabolic dysfunction-associated steatohepatitis, and increased liver stiffness in a general population setting. Am J Gastroenterol 2025.

Received June 30, 2025. Accepted October 3, 2025.

Correspondence:
Address correspondence to: Laurens A. van Kleef, MD, PhD, Department of Gastroenterology and Hepatology, Erasmus MC University Medical Centre, Rotterdam, the Netherlands. e-mail: l.vankleef@erasmusmc.nl.

Acknowledgments:
The authors gratefully acknowledge the contribution of the participants of the NHANES cohort, research assistants and facilitating personnel.

Authors' Contributions:
Laurens A. van Kleef: Data collection, study design, data analysis, writing of the manuscript, critical review of the manuscript, writing of the manuscript, approval of final version and approval of submission. Mesut Savas: Study design, data analysis, writing of the manuscript, critical review of the manuscript, writing of the manuscript, approval of final version and approval of submission. Maurice Michel: Study design, data analysis, writing of the manuscript, critical review of the manuscript, writing of the manuscript, approval of final version and approval of submission. Cyrielle Caussy: Critical review of the manuscript, writing of the manuscript, approval of final version and approval of submission. Jesse Pustjens: Critical review of the manuscript, writing of the manuscript, approval of final version and approval of submission. Adriaan G. Holleboom: Critical review of the manuscript, writing of the manuscript, approval of final version and approval of submission. Elisabeth F. C. van Rossum: Critical review of the manuscript, writing of the manuscript, approval of final version and approval of submission. Harry L.A. Janssen: Critical review of the manuscript, writing of the manuscript, approval of final version and approval of submission. Jörn M. Schattenberg: Critical review of the manuscript, writing of the manuscript, approval of final version and approval of submission. Willem P. Brouwer: Data collection, study design, data analysis, writing of the manuscript, critical review of the manuscript, writing of the manuscript, approval of final version and approval of submission.

Conflicts of Interest:
These authors disclose the following: Maurice Michel receives speaker honorarium from Ipsen, NovoNordisk, and Gilead Sciences. Adriaan G. Holleboom received research grants from Gilead and Novo Nordisk, acted as consultant for Gilead, Echosens, Novo Nordisk, Norgine, Julius Clinical, Inventiva and Boehringer Ingelheim, (co)leads the LEGEND trial with Inventiva and the SYNCH trial with Caelus Health and the Akkermansia Company and participates in trials of 89BIO, Boehringer Ingelheim, Novo Nordisk, and Inventiva. Elisabeth F.C. van Rossum has previously been involved in the medical care for patient participating in a clinical trial from Rhythm Pharmaceuticals, Inc. (no personal funding) and received personal royalties for the lay book FAT the secret organ. Harry L.A. Janssen received grants from AbbVie, Arbutus, Gilead Sciences, Janssen and Roche, and is a consultant for Arbutus, Arena, Enyo, Gilead Sciences, GlaxoSmithKline (GSK), Janssen, Merck, Roche, Vir Biotechnology Inc. and Viroclinics. CC declares consultant fees from Gilead, NovoNordisk, AstraZeneca, Lilly, E-scopics, MSD, Bayer, Corcept and Echosens, grant support from Gilead. Jörn M. Schattenberg declares consultant honorary from Akero, Alentis, Alexion, Altimmune, Astra Zeneca, 89Bio, Bionorica, Boehringer Ingelheim, Gilead Sciences, GSK, HistoIndex, Ipsen, Inventiva Pharma, Madrigal Pharmaceuticals, Kríya Therapeutics, Lilly, MSD Sharp & Dohme GmbH, Nordic Bioscience, Northsea Therapeutics, Novartis, Novo Nordisk, Pfizer, Roche, Sanofi, Siemens Healthineers, Summit Clinical, and Vantage Biosciences Research; speaker honorarium from AbbVie, Boehringer Ingelheim, Gilead Sciences, Ipsen Novo Nordisk, Madrigal Pharmaceuticals, Worldwide Clinical Trials, Stockholder options: Hepta Bio. Willem P. Brouwer received speakers fees for Eli Lilly, is part of the advisory board of Novo Nordisk, and participates in trials of 89BIO, Boehringer Ingelheim, Novo Nordisk, and Inventiva Pharma. The remaining authors disclose no conflicts.

Funding:
Financial support was provided by the Foundation for Liver and Gastrointestinal Research, Rotterdam, the Netherlands. The funding source did not influence the study design, data collection, analysis and interpretation of the data, nor the writing of the report and decision to submit for publication.

Ethical Statement:
NHANES procedures and protocols were approved by the National Center for Health Statistics Research Ethics Review Board. Participants of the NHANES 2017-2020 cycle provided informed consent. This study was conducted according to the principles outlined in the Declaration of Helsinki and Istanbul.

Data Transparency Statement:
Data are publicly available from the NHANES database (https://www.cdc.gov/nchs/nhanes/index.htm).

Reporting Guidelines:
STROBE.

Supplemental information

Weight Change is Associated With Metabolic Liver Health in a General Population Extending Beyond Weight Loss Targets of International Guidelines

Laurens A. van Kleef, Mesut Savas, Maurice Michel, Cyrielle Caussy, Jesse Pustjens, Adriaan G. Holleboom, Elisabeth F.C. van Rossum, Harry L.A. Janssen, Jörn M. Schattenberg, and Willem P. Brouwer

Weight change is associated with metabolic liver health in a general population extending beyond weight loss targets of international guidelines

Laurens A. van Kleef[1], Mesut Savas[2,3] *, Maurice Michel[4,5] *, Cyrielle Caussy[6,7,] Jesse Pustjens[1], Adriaan G. Holleboom[8,9], Elisabeth F.C. van Rossum[2,3], Harry L.A. Janssen[1,10], Jörn M. Schattenberg[4,5] and Willem P. Brouwer[1]

* Indicates shared authorship

1. Department of Gastroenterology and Hepatology, Erasmus MC, University Medical Center, Rotterdam, The Netherlands
2. Department of Internal Medicine, Division of Endocrinology, Erasmus MC, University Medical Center, Rotterdam, The Netherlands
3. Obesity Center CGG, Erasmus MC, University Medical Center, Rotterdam, The Netherlands
4. Department of Internal Medicine II, Saarland University Medical Center, Homburg, Germany
5. Saarland University, Saarbrücken, Germany
6. Hospices Civils de Lyon, Département Endocrinologie, Diabète et Nutrition, Hôpital Lyon Sud, 69495 Pierre-Bénite, France
7. Univ Lyon, CarMen Laboratory, INSERM, INRA, INSA Lyon, Université Claude Bernard Lyon 1, 69495 Pierre-Bénite, France
8. Department of Vascular Medicine, Amsterdam UMC, The Netherlands.
9. Amsterdam Gastroenterology Endocrinology and Metabolism Institute, Amsterdam UMC, The Netherlands
10. Toronto Centre for Liver Disease, Toronto General Hospital, University Health Network, Canada

SUPPLEMENTARY DOCUMENTS

Supplementary Table 1: Participant characteristics

	≥3 % weight loss	stable weight	≥3% weight gain
n	1877	2957	1968
**Demographics**			
Age	50 [33, 63]	53 [38, 64]	40 [26, 56]
Male	919 (49.0)	1576 (53.3)	830 (42.2)
Ethnicity			
Asian	174 (9.3)	479 (16.2)	213 (10.8)
Black	536 (28.6)	717 (24.2)	552 (28.0)
Hispanic	450 (24.0)	599 (20.3)	522 (26.5)
Other	129 (6.9)	117 (4.0)	105 (5.3)
White	588 (31.3)	1045 (35.3)	576 (29.3)
**Comorbidity**			
Weight status (1-year prior)			
BMI ≥ 30 kg/m^2	1081 (58.5)	929 (31.9)	553 (28.6)
BMI 25-30 kg/m^2	505 (27.3)	981 (33.7)	602 (31.2)
BMI < 25 kg/m^2	262 (14.2)	998 (34.3)	776 (40.2)
Diabetes	440 (24.3)	540 (19.0)	240 (12.6)
Hypertension	936 (53.1)	1480 (52.9)	782 (42.4)
High waist circumference	1112 (60.9)	1523 (52.6)	1231 (63.9)
**Biochemistry**			
AST	18 [15, 22.25]	19 [16, 24]	19 [16, 24]
ALT	17 [13, 24]	18 [13, 26]	18 [13, 27]
HDL	1.4 (0.4)	1.4 (0.4)	1.4 (0.4)
Triglycerides	1.2 [0.9, 1.8]	1.3 [0.9, 1.9]	1.2 [0.9, 1.9]
**Outcomes**			
MASLD	774 (41.2)	1246 (42.1)	850 (43.2)
At-risk MASH	112 (6.4)	162 (5.9)	140 (7.6)
LSM ≥ 8 kPa	166 (9.2)	262 (9.1)	179 (9.3)
CAP	263 (62)	264 (62)	266 (62)
LSM	5.1 [4.2, 6.2]	5.0 [4.1, 6.1]	4.9 [4.0, 6.1]

Data is presented as mean (SD), median [P25-P75] or n and percentage.
Abbreviations: ALT, alanine aminotransferase; AST, aspartate aminotransferase; CAP, controlled attenuation parameter; HDL, hypodensity lipoprotein; LSM, liver stiffness measurement; MASLD, metabolic dysfunction associated steatotic liver disease; MASH, metabolic dysfunction steatohepatitis.

Supplementary Table 2: Associations between continuous 1-year weight change (per 5%) with MASLD, at-risk MASH and increased LSM stratified for sex

	male			female		
	OR	95% CI	*P*	OR	95% CI	*P*
**MASLD**						
Weight gain	1.38	1.27 – 1.50	<0.001	1.17	1.10 – 1.24	< .001
Weight loss	0.66	0.60 – 0.72	<0.001	0.69	0.63 – 0.74	< .001
**At-risk MASH**						
Weight gain	1.20	1.10 – 1.31	<0.001	1.08	0.94 – 1.20	.247
Weight loss	0.81	0.70 – 0.93	0.003	0.79	0.66 – 0.93	.002
**LSM ≥ 8 kPa**						
Weight gain	1.22	1.12 – 1.33	<0.001	1.16	1.05 – 1.27	.002
Weight loss	0.84	0.73 – 0.94	0.005	0.76	0.66 – 0.86	< .001

Results were obtained with logistic regression models and given as OR with 95% CI for MASLD, at-risk MASH (based on FAST score ≥ 0.35) and LSM ≥ 8 kPa as outcome per 5% weight gain or 5% weight loss. The analysis included up to 3325 males and 3477 females. Results were adjusted in model 1 for age, ethnicity and weight (1 year ago). Abbreviations: CI, confidence interval; LSM, liver stiffness measurement; MASH, metabolic dysfunction associated steatohepatitis; MASLD, metabolic dysfunction associated steatotic liver disease; OR, odds ratio.

Supplementary Table 3: Associations between continuous 1-year weight change with MASLD, at-risk MASH and increased LSM for participants with ALT < 100 IU/L expressed per 5% weight change

	OR	95% CI	*P*
**MASLD**			
Weight gain	1.24	1.18 – 1.30	< .001
Weight loss	0.67	0.63 – 0.71	< .001
**At-risk MASH**			
Weight gain	1.14	1.06 – 1.23	< .001
Weight loss	0.78	0.70 – 0.88	< .001
**LSM ≥ 8 kPa**			
Weight gain	1.20	1.12 – 1.27	< .001
Weight loss	0.79	0.72 – 0.86	< .001

Results were obtained with logistic regression models and given as OR with 95% CI for MASLD, at-risk MASH (based on FAST score ≥ 0.35) and LSM ≥ 8 kPa as outcome per 5% weight gain or 5% weight loss. The analysis included up to 6753 individuals. Results were adjusted in model 1 for age, sex, ethnicity and prior weight (1 year). Abbreviations: CI, confidence interval; LSM, liver stiffness measurement; MASH, metabolic dysfunction associated steatohepatitis; MASLD, metabolic dysfunction associated steatotic liver disease; OR, odds ratio.

Supplementary Table 4: Associations between continuous 1-year weight change with MASLD, at-risk MASH and increased LSM for participants with daily ethanol intake < 20/30 gram in female and male expressed per 5% weight change

	OR	95% CI	*P*
**MASLD**			
Weight gain	1.24	1.18 – 1.30	< .001
Weight loss	0.67	0.63 – 0.71	< .001
**At-risk MASH**			
Weight gain	1.14	1.06 – 1.22	< .001
Weight loss	0.81	0.72 – 0.90	< .001
**LSM ≥ 8 kPa**			
Weight gain	1.19	1.12 – 1.27	< .001
Weight loss	0.79	0.72 – 0.87	< .001

Results were obtained with logistic regression models and given as OR with 95% CI for MASLD, at-risk MASH (based on FAST score ≥ 0.35) and LSM ≥ 8 kPa as outcome per 5% weight gain or 5% weight loss. The analysis included up to 6592 individuals. Results were adjusted in model 1 for age, sex, ethnicity and prior weight (1 year). Abbreviations: CI, confidence interval; LSM, liver stiffness measurement; MASH, metabolic dysfunction associated steatohepatitis; MASLD, metabolic dysfunction associated steatotic liver disease; OR, odds ratio.

Supplementary Table 5: Associations between continuous 1-year weight change with MASLD, at-risk MASH and increased LSM for participants trying to lose weight expressed per 5% weight change

	OR	95% CI	*P*
**MASLD**			
Weight gain	1.19	1.11 – 1.27	< .001
Weight loss	0.66	0.61 – 0.71	< .001
**At-risk MASH**			
Weight gain	1.10	0.99 – 1.21	.047
Weight loss	0.76	0.66 – 0.86	< .001
**LSM ≥ 8 kPa**			
Weight gain	1.23	1.13 – 1.33	< .001
Weight loss	0.72	0.64 – 0.81	< .001

Results were obtained with logistic regression models and given as OR with 95% CI for MASLD, at-risk MASH (based on FAST score ≥ 0.35) and LSM ≥ 8 kPa as outcome per 5% weight gain or 5% weight loss. The analysis included up to 3472 individuals. Results were adjusted in model 1 for age, sex, ethnicity and prior weight (1 year). Abbreviations: CI, confidence interval; LSM, liver stiffness measurement; MASH, metabolic dysfunction associated steatohepatitis; MASLD, metabolic dysfunction associated steatotic liver disease; OR, odds ratio.

Supplementary Figure 1: Weight loss and weight gain distribution

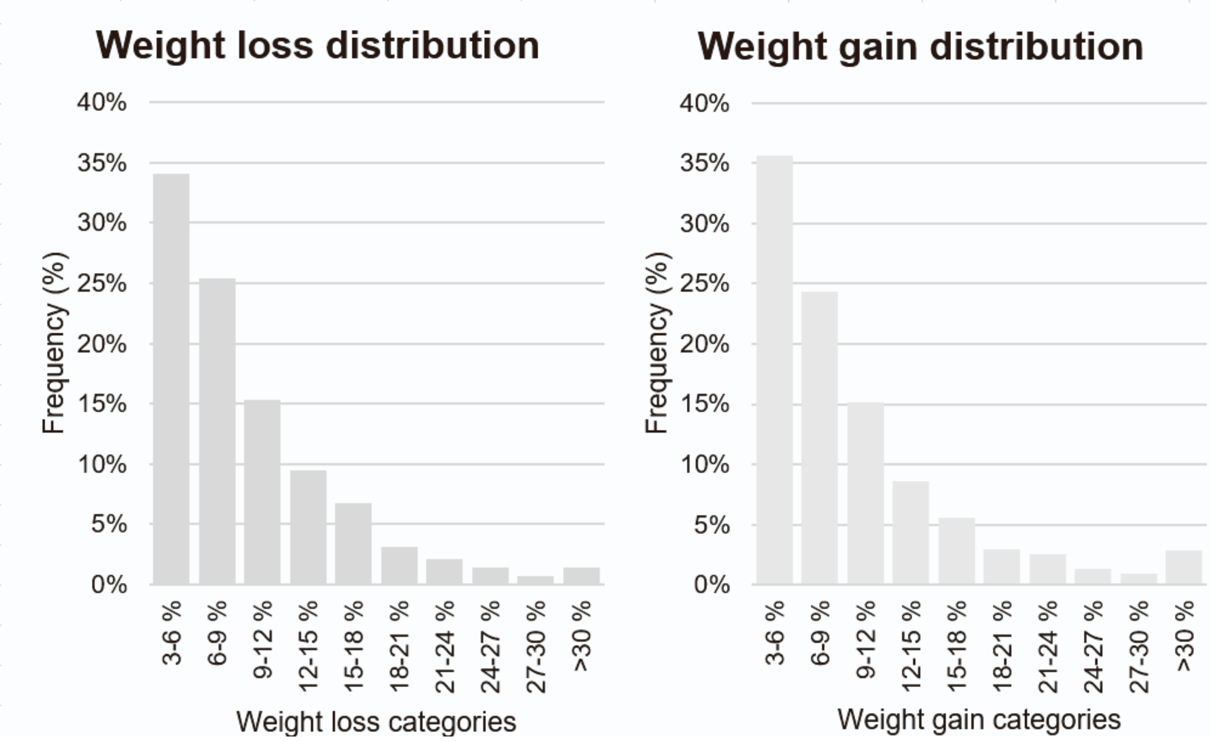

Weight loss of ≥ 3% was present in 1877 participants and weight gain of ≥ 3% in 1968.
